# Cellulose Composites with Graphene for Tissue Engineering Applications

**DOI:** 10.3390/ma13235347

**Published:** 2020-11-25

**Authors:** Madalina Oprea, Stefan Ioan Voicu

**Affiliations:** 1 Faculty of Applied Chemistry and Materials Science, University Politehnica of Bucharest, Gheorghe Polizu 1-7, 011061 Bucharest, Romania; madalinna.calarasu@gmail.com; 2 Advanced Polymer Materials Group, Faculty of Applied Chemistry and Material Science, University Politehnica of Bucharest, Gheorghe Polizu 1-7, 011061 Bucharest, Romania

**Keywords:** scaffolds, membranes, hydrogels, cellulose, tissue engineering

## Abstract

Tissue engineering is an interdisciplinary field that combines principles of engineering and life sciences to obtain biomaterials capable of maintaining, improving, or substituting the function of various tissues or even an entire organ. In virtue of its high availability, biocompatibility and versatility, cellulose was considered a promising platform for such applications. The combination of cellulose with graphene or graphene derivatives leads to the obtainment of superior composites in terms of cellular attachment, growth and proliferation, integration into host tissue, and stem cell differentiation toward specific lineages. The current review provides an up-to-date summary of the status of the field of cellulose composites with graphene for tissue engineering applications. The preparation methods and the biological performance of cellulose paper, bacterial cellulose, and cellulose derivatives-based composites with graphene, graphene oxide and reduced graphene oxide were mainly discussed. The importance of the cellulose-based matrix and the contribution of graphene and graphene derivatives fillers as well as several key applications of these hybrid materials, particularly for the development of multifunctional scaffolds for cell culture, bone and neural tissue regeneration were also highlighted.

## 1. Introduction

### 1.1. General Aspects Concerning Tissue Engineering

In recent decades, the rapidly aging population, environmental stressors, frequent cases of traumatic injuries and chronic diseases lead to a growing interest in the revolutionary domain of tissue engineering (TE) [1,2,3]. Tissue engineering evolved from the field of biomaterials; its purpose is to combine scaffolds, cells, and biologically active molecules to obtain multifunctional materials that restore, maintain or improve damaged tissues or an entire organ. Some examples of Food and Drug Administration (FDA)-approved engineered tissues include artificial skin and cartilage, but they have limited use in human medicine due to several yet unknown aspects regarding their long term biocompatibility [4,5,6,7,8,9]. Bioactive scaffolds, cell therapy, smart drug delivery systems, and wound healing mats are some representative examples of the research topics approached by TE. In addition to medical applications, non-therapeutic findings include the use of tissues as biosensors to detect chemical or biological threats or the development of organs-on-a-chip for toxicity screening of experimental medication [4,8,10].

Porous three-dimensional (3D) scaffolds are an important component of tissue engineering. These constructs are used to provide an appropriate environment for tissue and organs regeneration. Biological scaffolds (e.g., fibrin, amniotic membrane, and perfusion-decellularized organs) are an accessible option because they already contain a broad spectrum of signaling molecules with an important role in the processes of cellular morphogenesis and function development [11,12]. However, their composition is strongly related to their source of origin, therefore they have poor reproducibility. Biomaterials-based scaffolds have the advantage that they can be tailored to meet specific requirements, the result being a controllable environment in which stem cells and growth factors can be incorporated to recreate various tissues [4,5,13]. Considering the response of the body’s immune system, it is recommended that scaffolds replicate the native extracellular matrix (ECM) of different tissues, in terms of physical structure, chemical composition and biological functionality [13,14,15,16]. Biocompatibility, non-immunogenicity, and non-toxicity are mandatory features of biomaterials-based scaffolds. Their design and mechanical properties are also important because they should have the ability to enhance cell migration, proliferation, and differentiation, by presenting appropriate biomechanical, biophysical, and biochemical signals, in vivo, while maintaining their shape and integrity [17,18]. 

### 1.2. Characteristics Recommending Cellulose and Graphene for Applications in Tissue Engineering 

Cellulose remarks itself among the biomaterials used for scaffold production due to its high availability and renewability. Cellulose is mainly extracted from plant cell walls. The nano-scaled forms of plant cellulose—cellulose nanofibers (CNFs) and cellulose nanocrystals (CNCs), are obtained following specific mechanical and chemical treatments (e.g., ball milling, enzymatic or chemical hydrolysis, TEMPO-mediated oxidation) [3,19]. In virtue of its natural origin, cellulose has a native biocompatibility and negligible cytotoxicity [20]. Some issue were posed related to the inflammatory effect and oxidative stress caused by the cellular uptake of the nano-scaled forms of cellulose [21] but studies showed that both cellulose nanocrystals and cellulose nanofibers presented a non-immunogenic and non-cytotoxic character when different mammalian cell lines were exposed to CNCs suspensions [22] or CNFs membranes [23].

Cellulose can also be produced by certain microorganisms, in this case being called bacterial cellulose (BC). BC is considered to be the most biocompatible form of cellulose because it lacks biogenic impurities such as lignin and hemicellulose and only mild chemical treatments are required to ensure its purity [24]. The nanofibrillar porous structure of bacterial cellulose is similar to the extracellular matrix, this making BC one of the most recommended materials for tissue engineering scaffolds [14,25,26]. Previous studies highlighted that BC has the ability to reduce the inflammatory response and increase the rate of tissue regeneration when it is used as wound dressing [25,26,27]. Due to its excellent mechanical properties, BC was considered a promising material for the development of vascular grafts, dental implants, artificial skin, and blood vessels [25,28,29,30,31,32].

Pure cellulose lacks solubility in common organic solvents. This represents an issue when it comes to processing techniques where a stable polymeric solution is required to prepare the final material (e.g., electrospinning, phase inversion). Cellulose was dissolved so far only in mixtures of highly toxic solvents (e.g., ionic liquids, carbon disulfide, N-methyl-morpholyne-N-oxide, dimethylformamide) [33]; however, for biomedical applications such as tissue engineering, they are not recommended because even small traces of such solvents could cause a substantial biocompatibility decrease. Consequently, cellulose derivatives were developed, their improved dissolution ability in less noxious solvents, or even water, encouraging their use as an alternative to pure cellulose [3]. Moreover, cellulose derivatives maintain the biocompatibility features of pristine cellulose presenting mild or no foreign body reaction during in vivo assays [34].

**Figure 1:**
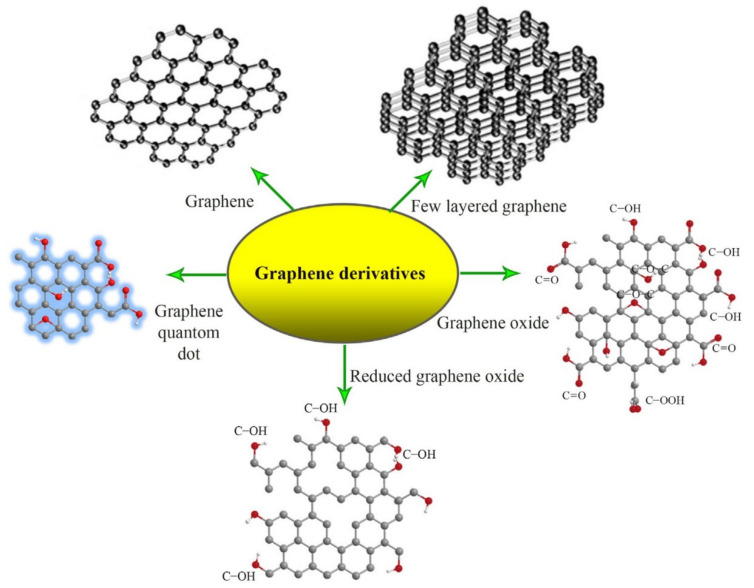
The chemical structures of graphene and its derivatives [46]. Reproduced with copyright permission.

Graphene (GE) is an allotrope of carbon produced by top down (e.g., mechanical or chemical exfoliation of graphite, chemical synthesis) or bottom up techniques (e.g., chemical vapor deposition, pyrolysis, epitaxial growth) [35]. GE has some unique properties such as high specific surface area, superior electrical and thermal conductivity, and excellent mechanical properties. The free π electrons and reactive sites, generated by the plane carbon-carbon bonds in GE’s aromatic structure, ensure it a facile surface functionalization [36,37]. However, the hydrophobicity and strong interactions between sheets hinder the dispersion of GE in aqueous or organic environments [38,39]. This issue was solved with the development of graphene derivatives such as graphene oxide (GO) and reduced graphene oxide (rGO) which possess specific surface groups that allow them to be effectively dispersed in a wide range of solvents or to be incorporated in polymeric matrices (Figure 1) [40,41]. For example, graphene oxide presents hydroxyl functional groups on the upper and bottom surface as well as carboxylic groups on the edges. This chemical structure is characterized by a hydrophilic character that enables GO’s dispersion in water and polar solvents and facilitates hydrogen bonding with polymeric matrices [42]. Reduced graphene oxide is characterized by a lower hydrophilicity and oxygen content but an enhanced electrical conductivity. It was showed that the addition of rGO in polymer composites can increase the thermal stability, improve the bioactivity and mechanical properties, and also provide an appropriate medium for electrical stimulation procedures [40,43,44]. A special type of graphene derivative is represented by graphene quantum dots (GQDs). This nano-scaled form of graphene is made up of one or a few GE layers, with lateral dimensions smaller than 10 nm (Figure 1). GQDs photoluminescence and quantum confinement effect recommend them for applications in bioimagistics, biosensors and photocatalysis devices [45]. 

Generally, graphene and its derivatives are considered biocompatible and non-cytotoxic, still, their preparation method highly influences the in vivo and in vitro tests because the residual solvents and reagents used during the synthesis procedures can interact with cells and tissues, thus inducing cytotoxicity and oxidative stress. The hydrazine used to obtain reduced graphene oxide was found to be particularly noxious for human mesenchymal stem cells (hMSCs) [47]. Eco-friendly reduction methods were developed to diminish these residual chemicals-induced adverse effects. For example, Erdal et al. used a microwave-induced hydrothermal reaction and caffeic acid (Caf), a green reducing agent, to produce nanosized reduced graphene oxide (n-rGO), starting from commercial α-cellulose. Cellulose was treated with an aqueous solution of H_2_SO_4_ in a microwave device. The material was kept at 180 °C and a pressure of 40 bar, for 2 h, under nitrogen flow. Following this treatment, black solid carbon spheres were obtained. The spheres were further dispersed in concentrated nitric acid (HNO_3_), ultrasonicated 30 min at 45 °C and heated at 90 °C for 30 min, under magnetic stirring to obtain nGO. The ultrasonication time is a decisive factor in obtaining materials with uniform and reproducible properties [48]. The green reduction process was performed by placing an aqueous suspension of nGO and Caf in the microwave device, using the same conditions as in the case of carbon spheres synthesis [49]. The resulting n-rGO was incorporated in polycaprolactone (PCL) matrices for the production of bioactive and bioresorbable composites [50], 3D scaffolds with drug delivery ability [51] and macroporous scaffolds with applications in bone tissue engineering [52]. 

The size and oxidation status of GE are also important for cytotoxicity evaluation. GE and GE derivatives may disrupt cell membranes by direct contact. Moreover, small sized GO has a high potential of being internalized into cells via endocytosis and could cause apoptosis at high concentrations [53]. Surface modification with biocompatible molecules or the insertion in biopolymer matrices were the main solutions proposed to minimize the potential cytotoxic character of GE and GE derivatives. In addition, after incorporation in a polymer matrix, the carbonaceous fillers provide cellular binding sites and, in the case of GO, the oxygenated surface groups increase the hydrophilic character, thus improving cellular adhesion [47]. 

GE and its derivatives have a demonstrated ability to promote stem cells differentiation processes, particularly adipogenesis and osteogenesis by enhancing the adsorption of differentiation factors and cell adhesion [54]. Graphene-induced osteogenesis was found to be related to the activation of the mechanosensitive integrin-focal adhesion kinase (FAK) axis and also to GE’s capacity of promoting the paracrine release of pro-osteogenic molecules in its surroundings, as well as enhancing their delivery to the sites of action [55]. According to recent studies, graphene oxide and reduced graphene oxide are pro-angiogenic. The mechanisms for GO and rGO induced angiogenesis include intracellular formation of reactive oxygen and nitrogen species as well as activation of specific serum antibodies (e.g., phospho-eNOS, phospho-Akt) [56]. The potential of reduced graphene oxide (rGO) to enhance angiogenesis was evaluated by Chakraborty et al. using polyvinyl alcohol/carboxymethyl cellulose (PVA/CMC) scaffolds loaded with different concentrations of rGO nanoparticles. Primary biocompatibility studies included in vitro alamarBlue cytotoxicity assays on three different cell lines—fibroblasts NIH3T3, endothelial-like cells (ECV304) and endothelial cells (EA.hy926). The scaffolds showed no toxicity toward the analyzed cell lines, the cellular viability being similar to the control group. It was concluded that when incorporated inside a scaffold, rGO does not present cytotoxicity even if it is used in concentrations higher than the cytotoxicity threshold in free solution (100 ng/mL). The composite scaffolds were implanted in chick chorioallantoic membrane (CAM) models to study their influence on the neovascularization process. Two days following implantation, the number and wall thickness of the blood vessels were substantially increased, compared to the untreated control. Moreover, angiogenesis and arteriogenesis were enhanced as the rGO concentration in the composites increased, whereas neat PVC/CMC scaffolds showed no bioactivity [57]. 

As a results of their remarkable properties, cellulose/graphene composites were extensively researched particularly for biomedical applications. Cellulose is a versatile, highly available, biodegradable, and biocompatible material, these characteristics recommending it as a low cost, sustainable alternative to petroleum-based plastics or other types of natural polymers used in tissue engineering. Cellulose/graphene composites designed for the TE field can be divided in two main categories—composites where specific types of cellulose (e.g., cellulose paper [58], bacterial cellulose [59], cellulose derivatives [60]) are employed as polymer matrices in which the carbonaceous fillers are dispersed to improve their mechanical characteristics and biocompatibility, or, composite fillers based on CNCs or CNFs combined with GE or GE derivatives that are used to synergistically reinforce other polymer matrices [21] (e.g., polylactic acid—PLA [61,62], polybutylene succinate—PBS [63], polyacrylamide—PAM [38], polycaprolactone—PCL [64]). Composite membranes with graphene [65,66] were also studied for applications in the hemodialysis field [67,68]. The use of GO for reinforcing cellulose acetate membranes led to an increase of bovine serum albumin (BSA) retention from 80% to over 96% [69,70]. The synergistic effect between GO and carbon nanotubes (CNT) used for the preparation of composite cellulose acetate membranes with potential applications in hemodialysis showed good results in the retention of BSA and hemoglobin [71]. These results are due to both the presence of GO, which has a high surface adsorption ability of the proteins that need to be separated [72,73], and also to the weak chemical interactions that emerge between the delocalized electrons on the surface of graphene and the non-participating electrons from the functional groups of the polymer [74,75]. 

The enumerated types of cellulose/graphene composites were discussed in detail in the following sections of this review.

## 2. Cellulose Composites with Graphene for Tissue Engineering Applications

An ideal material for tissue engineering should have an excellent biocompatibility with the cellular components and mimic, as much as possible, the extracellular matrix of the recreated tissue. A porous structure with interconnected porosity is among the essential characteristics of a scaffold in order to allow cellular attachment and migration and facilitate the diffusion of nutrients and metabolites throughout its volume [17]. Moreover, the mechanical characteristics must not be neglected because they should match the ones of the host tissue. Studies showed that both stem and mature cells are sensitive to the stiffness of the substrate on which they are seeded and show different adhesion and morphological characteristics depending on it. The scaffolds should also possess a certain degree of bioactivity and interact with the surrounding environment to actively regulate cellular activity [76]. The recreated tissue type dictates the design and functionality of TE scaffolds. For example, in the case of bone tissue engineering, the restoration of normal biomechanical functions is crucial, therefore, the scaffolds must have similar mechanical properties to the native bone and a degradation behavior matching with the novel bone formation rate [77]. Electrical properties are another important aspect, especially for neural tissue engineering, because the neurons proliferation, migration and communication with other cell types is realized by electrical signaling mechanisms [78]. Furthermore, electrical stimulation performed during cell culture was showed to enhance cell migration, proliferation rate and differentiation, being considered a revolutionary tool for tissue engineering and regenerative medicine [79]. 

In the next sections of this review, the fabrication methods, physico-chemical features and biological performance of cellulose/graphene composites for tissue engineering will be described. As it will be observed, by carefully choosing the type of cellulose and graphene used as well as the preparation technique, it is possible to obtain scaffolds with tunable characteristics depending on the desired application.

### 2.1. Cellulose Paper-Graphene Composites

Paper-based scaffolds were first developed starting from surgical grade cotton or bacterial cellulose. The cotton-derived substrates were obtained by a simple and cheap paper making process involving cooking and beating the cotton before pressing it in a British sheet forming device. The resulting paper was afterwards immersed in a gelatin solution to enhance its cellular adhesion ability. According to the FESEM and MTT assay, MG63 cells incubated on all of the developed scaffolds presented a normal morphology but the adhesion and proliferation rates increased on the gelatin-modified ones [80]. Cheng et al. used bacterial cellulose and hydrophobic petroleum jelly-liquid paraffin ink to create cost effective tissue models (~4 cents per single device) by the matrix assisted sacrificial 3D printing technique. The fugitive ink was 3D printed on the wet BC pellicles in a predetermined pattern. The pellicles were air dried and then heated at 70 °C to liquefy the ink and remove it by rinsing with n-hexane and distilled water, thus resulting well-defined microchannels inside the cellulosic matrix. The obtained devices were tested as vascularized breast tumor models by seeding green fluorescence protein (GFP)-labeled human umbilical vein endothelial cells (HUVECs) inside the microchannels and MCF-7 cells on the surrounding cellulosic matrix. The drug response of the tissue model was also evaluated by injecting tamoxifen in the endothelialized microchannels [81]. 

**Figure 2:**
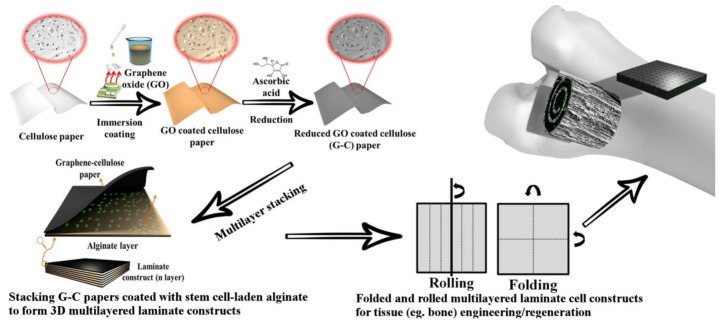
The fabrication stages of G-C paper and origami inspired cell-laden constructs (**left**); Rolling and folding of the 3D structures for applications in bone tissue regeneration (**right**) [58]. Reproduced with copyright permission.

Graphene-enriched scaffolds improve stem cell viability and osteogenesis by participating in the activation of physiologically relevant mechano-transduction pathways. GE’s topographical features and electrical conductivity facilitate cell anchorage and hydroxyapatite formation ability with physio electrical signal transfer, thus enhancing osteogenesis [64,82]. A novel graphene-cellulose (G-C) paper scaffold was developed by Li et al. for applications such as in vitro modeling of human bone development and regeneration, or bone patches and plugs to facilitate in vivo osteogenesis following injuries. Commercial tissues, blotting paper and filter paper were tested as substrates for the fabrication of G-C composites. The papers were laser cut to 1 cm × 1 cm size and aqueous dispersions of GO were deposited on the substrates. The resultant GO-coated papers were dried at 100 °C for 2 min and reduced in 50 mM L-ascorbic acid solution at 80 °C for 3 h (Figure 2 left) [58]. It was found that the G-C papers electrical conductivity and mechanical properties were positively influenced by the rGO and could be tuned by modifying the number of deposited rGO layers. The composites presented an improved biocompatibility, translated by a higher surface live cell density, compared to the uncoated paper. This was attributed to an increase in hydrophilicity caused by rGO addition that favored human adipose derived stem cells (hASDCs) adhesion. Higher levels of alkaline phosphatase (ALP) were observed for the G-C papers-cultured cells, this suggesting that rGO also guided the cellular differentiation into an osteogenic lineage.

An interesting fact was that the G-C papers could be laminated with alginate and folded or rolled to obtain origami inspired cell-laden constructs as shown in Figure 2 (right). Similar cellulose paper/cell-laden structures were previously obtained using commercial chromatography papers and HS-5 human bone marrow stromal cells suspended in Matrigel [83] or filter papers and MDA-MB-231-GFP breast cancer cells suspended also in Matrigel [84]. In another study, sodium alginate containing 3T3 mouse fibroblasts was used as bioink to create ultrafine patterns on chromatography papers treated with an alginate crosslinking solution (CaCl_2_) [85]. However, in this studies, cellulose was employed just as a support for the cell-embedded hydrogels, compared to the G-C papers where the modified cellulose itself played an important role in cellular growth and proliferation. The 3D structures were obtained by suspending hASDCs in an alginate solution followed by drop-wise deposition of a small amount of hADSCs-laden alginate on the upper surface of each G-C paper and stacking of multiple such sheets before crosslinking them by immersion CaCl2 (Figure 2 left). Cross sectional SEM images showed that these 3D constructs had a stratified structure, the alginate hydrogel effectively binding the G-C papers. Viable cells were observed at the hydrogel-paper interface immediately post assembly and after a 42 days study period, therefore, it was concluded that the 3D G-C-paper/alginate structures are able to provide a long term cellular support [58]. 

**Figure 3:**
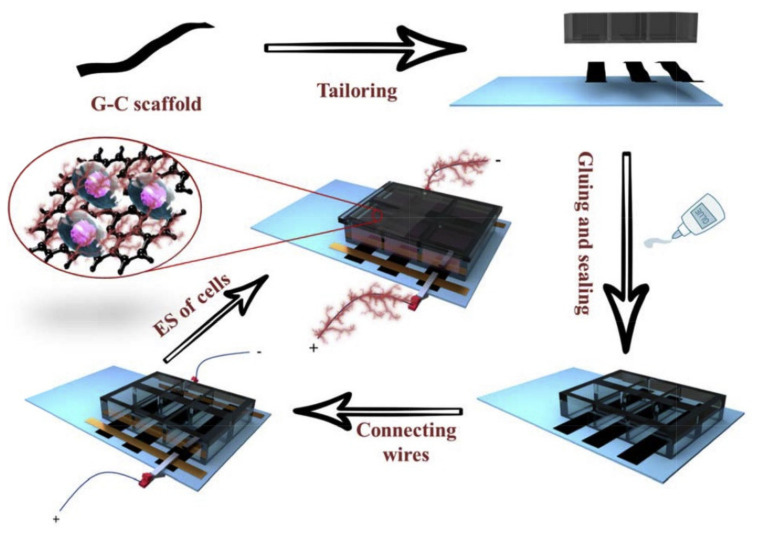
Schematic representation of the assembly and functionality of a G-C-based electrode for cellular electrostimulation [82]. Reproduced with copyright permission.

The developed G-C papers also have the potential to be used for cellular electrical simulation (ES) due to the electrical conductivity provided by the incorporated rGO. This subject was thoroughly analyzed by the same research group. The study was made by integrating the electroactive paper in polystyrene (PS) chambers to obtain electrodes. The electrode assembly process is illustrated in Figure 3. Briefly, the G-C papers were cut into strips and mounted in parallel on a glass substrate. The PS chamber, with an open bottom and removable lid, was attached on top of the glass substrate using silicone and copper tapes were glued on both edges, perpendicular to the G-C strips, to connect them. The electrode was afterwards coupled to an electrical simulator using copper wires [82].

During electrochemical characterization by cyclic voltammetry and electrochemical impedance spectroscopy, the G-C electrodes showed high stability, lower impedance, and higher charge injection capacity than commercial gold electrodes. hADSCs were cultured on the G-C scaffolds with or without electrical stimulation. At the end of the 28 days study period it was found that electrically simulated cells showed increased proliferation, mineral deposition and ALP expression compared to control samples. According to these results, it was considered that the developed G-C electrodes could represent an alternative to conventional metal electrodes that present the risk of corrosion-related cell compatibility issues [82]. 

### 2.2. Bacterial Cellulose-Graphene Composites

**Figure 4:**
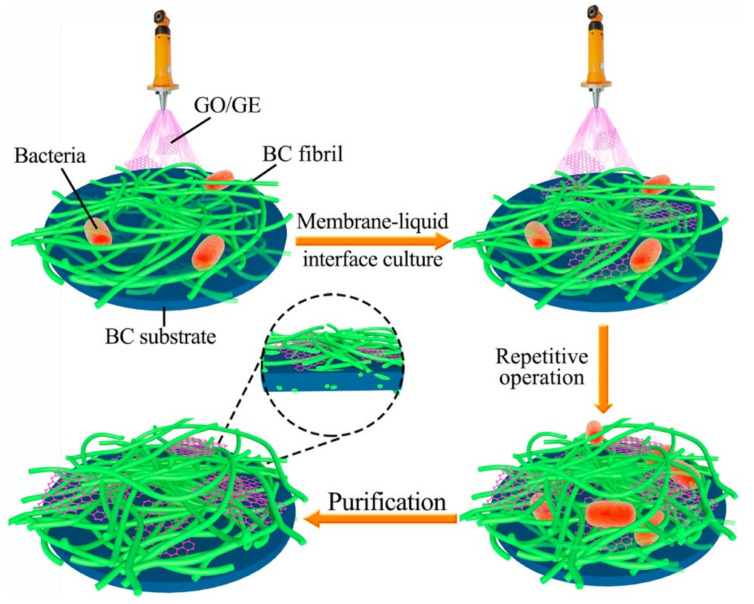
Schematic representation of the steps involved in the MILIC fabrication method used for the GO/BC and GE/BC scaffolds [87]. Reproduced with copyright permission.

Bacterial cellulose/graphene composites (BC/GE) represent an intensively researched subject in tissue engineering. There are two essential requirements for the successful preparation of BC-based composites with GE—the finding of an appropriate synthesis method that maintains the intrinsic nanofibrous structure of BC and the homogenous dispersion of the carbon-based material in the cellulosic matrix [86]. Both graphene and graphene oxide are promising materials for biomedical applications, still, recent studies suggest that the incorporation of GE and GO into 3D nanofibrous scaffolds results in different biological properties, and GO-based scaffolds have a better biocompatibility and bioactivity compared to the ones containing GE [87]. GE and GO-reinforced BC scaffolds were prepared using an accessible membrane-liquid interface culture (MILIC) method. The MILIC technique consisted of the pulverization of a GE or GO-containing culture medium onto BC pellicles obtained from conventional static cultures. A thin layer of GE/BC or GO/BC was formed on the neat BC surface and served as a new substrate for the next hydrogel layer as illustrated in Figure 4. The process was repeated until a desired thickness was reached. After purification and freeze drying, the morphologies, structure, mechanical properties and biocompatibility of BC/GE and BC/GO were compared between them and with pristine BC scaffolds as control.

Cellular viability assays were conducted using mouse embryonic osteoblasts (MC3T3-E1). As expected, BC/GO scaffolds displayed a better cellular adhesion, spreading, proliferation and osteogenic differentiation compared to neat BC and BC/GE. A possible explanation could be that GO’s hydrophilic surface groups provide an improved cytocompatibility [87,88]. 

Bacterial cellulose/reduced graphene oxide (rGO) films were also studied for applications in biomedical device fabrication, biosensors, and tissue engineering. The composites were obtained using a bacteria-mediated reduction technique. Gluconacetobacter intermedius (BC 41) was cultured in a mixture of culture medium and GO in a static incubator. During the 14 days culture period, BC/GO composites self-assembled in situ and the GO on the surface of the cellulose fibers was biochemically reduced. Cross section SEM images revealed an interconnected structure comprised of stacked rGO sheets linked together by BC nanofibrils. This distinct structure was associated with the favorable mechanical characteristics of the composites, mechanical resistance being a characteristic required to ensure their functionality under the harsh conditions in living organisms. The electrochemical performance of rGO obtained by bacterial reduction was inferior to chemically synthesized rGO; however, it still exhibited a high charge carrying capacity at a given voltage and was considered sufficient to be used for cellular electrical simulation or to collect physiological signals in biosensors. Human marrow mesenchymal stem cells (HMSCs) were used to monitor the cellular response of the BC/rGO films in terms of cellular adhesion and proliferation. Tissue culture polystyrene (TCP) and plain rGO films were set as control groups. After 7 days in culture, HMSCs were present in a higher number on BC/rGO substrates (9.65 × 104) compared to the control groups (6.89 × 104—rGO and 9.15 × 104—TCP). Also, BC/rGO-grown cells displayed better cell attachment and retention when observed using confocal microscopy after 3 days of culture on the analyzed substrates [89]. 

For enhanced bioactivity, graphene oxide was coated with hydroxyapatite (HA) by a wet chemical precipitation route. Briefly, calcium hydroxide and ortho-phosphoric acid were incorporated in a GO solution (1 mg/mL concentration) by magnetic stirring. The residue was aged for 24 h, washed with distilled water and dried in a hot air oven. The resulting GO-HA complex was incorporated in bacterial cellulose matrices by impregnating the wet BC membrane with the GO-HA suspension in ethanol, under continuous stirring for 24 h. Due to the osteoinductive and osteoconductive properties of hydroxyapatite [90], GO-HA could ensure a higher viability of osteoblasts compared to unmodified GO. The biological characterization was performed on normal (NIH3T3) and osteosarcoma (MG-63) cell lines, and consisted of methyl thiazolyl tetrazolium (MTT) assay and alkaline phosphatase (ALP) activity measuring in culture supernatants. The viability of both MG-63 and NIH3T3 cells in the presence of BC/GO-HA composites was better compared to BC, GO and BC/HA and a higher ALP activity was observed, particularly at a concentration of 50 µg/mL. The results were confirmed by the phase-contrast microscopy images that showed an increased cell density on the BC/GO-HA composites surface compared to the other materials tested [91]. 

Another area where bacterial cellulose/graphene composites showed promising results is represented by neural tissue engineering. The use of bacterial cellulose/graphene-based scaffolds for cell-based regenerative therapy could solve the main problems associated with this technique, more specifically, the decreased cellular viability, poor integration within the host brain tissue and decreased tendency of implanted stem cells to differentiate toward functional neurons. For an effective reconstruction of injured brain tissues, the used scaffold should reduce inflammation and apoptosis, promote restorative processes, neurite outgrowth as well as axonal elongation [92]. A first attempt to obtain such constructs was made by Si et al. The research group developed BC/GO nanocomposite hydrogels using a facile one-step in situ biosynthesis. A commercial aqueous dispersion of GO nanosheets was added to the BC culture medium, followed by intense stirring for 60 min. The resulting BC/GO pellicles were soaked in deionized water at 90 °C for 2 h and boiled in sodium hydroxide (NaOH) solution for 15 min for purification. The materials were then washed until they reached a neutral pH. SEM images revealed that the GO nanosheets were uniformly dispersed within the BC matrix and the 3D fibrous network and porous structure of BC was kept after the incorporation of the inorganic compounds. The good structural properties of BC/GO composites were attributed to the in situ biosynthesis method that favored strong interactions between the hydroxyl groups on both BC and GO surface. Also, GO maintained its crystal structure the characteristic crystal lattices being visible in TEM images. Tensile testing of BC/GO composites was performed using a universal material testing instrument, under ambient temperature and humidity. The inclusion of GO in the BC structure lead to a notable increase of the tensile strength and Young modulus, as showed by the stress-strain curves. This improvement was considered the result of the strong interfacial interactions and homogenous dispersion of the carbonaceous filler inside the polymeric matrix, which favored an effective load transfer from BC to GO [86].

**Figure 5:**
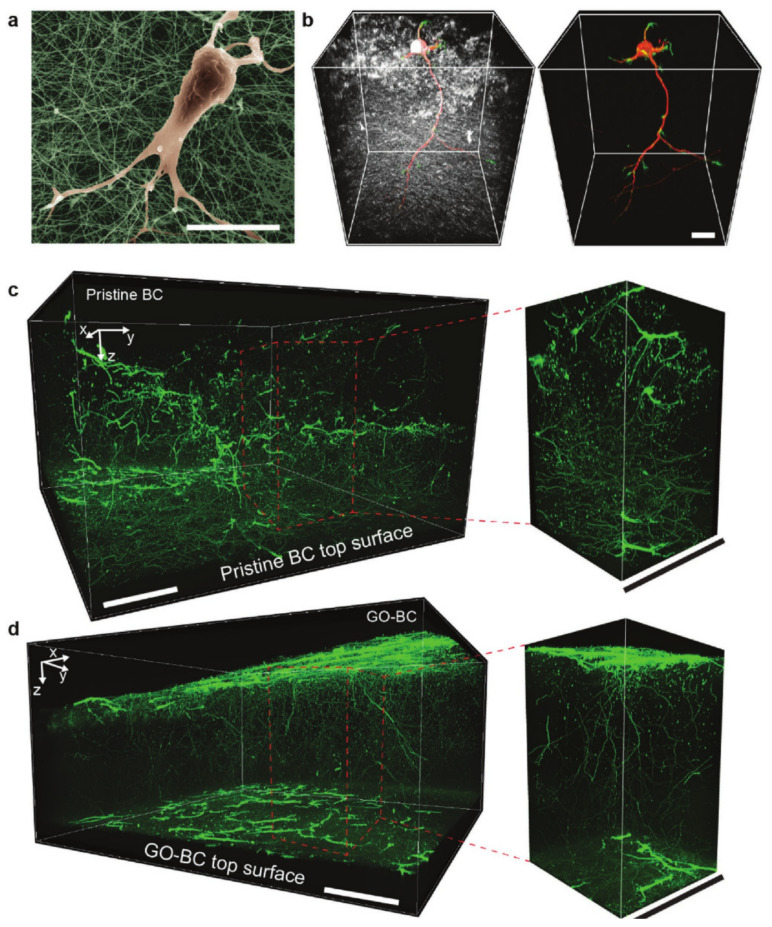
Pseudo-colored SEM image of an E18 neuron seeded on neat BC (**a**) 3D confocal fluorescence images of an E18 neuron development inside neat BC scaffolds (**b**) 3D confocal fluorescence images of Tuj1 red and Phalloidin green-stained neurons cultured on neat BC (**c**) and BC/GO hydrogels (**d**) [93]. Reproduced with copyright permission.

Further studies on biosynthesized BC/GO scaffolds were conducted by Kim et al. They reported a non-genetic manipulation method of Acetobacter xylinum that resulted in the synthesis of carbon-hybridized BC hydrogels. The bacteria was incubated for 14 days in a culture medium containing GO stabilized with a comb-like amphiphilic polymer (APCLP) for a better dispersion. The resulting composites were purified with NaOH, washed with distilled water and vacuum dried before characterization. The crystallinity of the hybrid BC/GO scaffolds was lower compared to neat BC while the porosity was higher. These characteristics were associated with the accelerated formation of the BC pellicles, and the perturbation of individual polysaccharide chains crystallization in the culture medium containing APCLP-GO, which lead to a densification of the cellulose nanofibrils, thus increasing the overall porosity. For the biological assessment, a neuronal network was constructed by seeding rat embryonic hippocampal neurons (E18) (Figure 5a) on both sides of the synthesized scaffolds and neat BC pellicles for comparison purpose. As the neurons developed inside the scaffolds (Figure 5b), their terminations interconnected vertically, thus forming a long range neuronal network referred as “minibrain” (Figure 5c,d). Neurons cultured on BC and BC/GO substrates had an accelerated neuronal processes development, compared to the ones cultured on conventional flat glass substrates that presented only early stage dendrites expansion. These differences were attributed to the nanofibrillar ECM-like structure of BC that may simulate cellular development and even guide neurite pathfinding [93].

No significant differences in embryonic rat hippocampal neurons development were observed between neat BC and BC/GO. The general conclusion was that GO had little or no influence and the accelerated growth of the neuronal processes (compared to conventional glass substrates) was mainly attributed to the fibrous structure of BC. However, in a different study, neural development was studied using human neural stem cells and the results indicated that GO had an important role in guiding and accelerating the cellular differentiation process toward the neuronal lineage and also enhanced neurites formation, elongation, and branching. The study was conducted by Park et al. that developed 3D hybrid scaffolds based on bacterial cellulose and amphiphilic comb-like polymer (APCLP)-covered graphene oxide flakes, and applied them as brain cortex mimetics in motor cortectomy rat models [92]. The GO flakes, prepared by a chemical exfoliation process (Hummers method), were coated with APCLP for a uniform dispersion, and added in the bacterial culture medium. The obtained membranes were purified with NaOH, washed with distilled water, and dried overnight at 60 °C. Human neural stem cells (F3), isolated from embryonic brains, were seeded within the BC and BC/GO-APCLP scaffolds. The cellular development was observed via phase-contrast microscopy. It was observed that two days after seeding, long neurites started growing from the BC/GO-APCLP-cultured F3 cells whiles the BC-cultured ones showed no substantial changes in morphology. Immunofluorescence assays were further employed for a better understanding of the cellular proliferation and differentiation processes occurring in the scaffolds. Luciferase activity indicated that the number of F3 cells increased constantly until the 8th day of the study but mature neuronal markers (MAP2) and synaptic vesicle proteins (synaptophysin), were present only in BC/GO-APCLP scaffolds. The cell-enriched scaffolds were implanted to motor cortex-ablated rats with mimicked trauma injuries and the cellular behavior using in vivo molecular imaging. According to the bioluminescence signals, representing the number of viable F3 cells, the cells cultured on BC/GO-APCLP and neat BC scaffolds had a higher survival rate (12 days vs. 10 days) compared to the cell-only treated group (conventional cell therapy). After the test period, excised brains were analyzed by immunohistochemistry. Similar to the in vitro tests, most of the cells cultured on BC/GO-APCLP scaffolds showed strong homogenous staining on MAP2 and synaptophysin. Only a few BC-cultured cells presented the same characteristics respectively no cells in the case of the control (cell-only treated) group [92]. 

### 2.3. Cellulose Derivatives-Graphene Composites

Electrospinning is a frequently used technique for the production of ECM-mimicking fibrous structures. The electrospinning device uses a high tension source to create an electrical field that draws charged polymer droplets from the tip of a needle to a collector plate, thus resulting micro and nano-scaled fibers [94,95]. In virtue of their native biocompatibility and surface topography that promotes cellular adhesion and influences the conformation of adsorbed adhesion proteins (e.g., fibronectin, vitronectin), electrospun cellulose-based fibers could be worthy candidates for the production of tissue engineering scaffolds [96,97]. Among cellulose derivatives, cellulose acetate (CA), in particular, showed good fiber forming ability in a variety of solvents, flexibility and excellent mechanical strength in fibrous form [98]. Cellulose acetate-based scaffolds have the ability to support osteoblasts growth, phenotype retention and bone formation [99], this encouraging their application as scaffolds for bone tissue engineering. 

**Figure 6:**
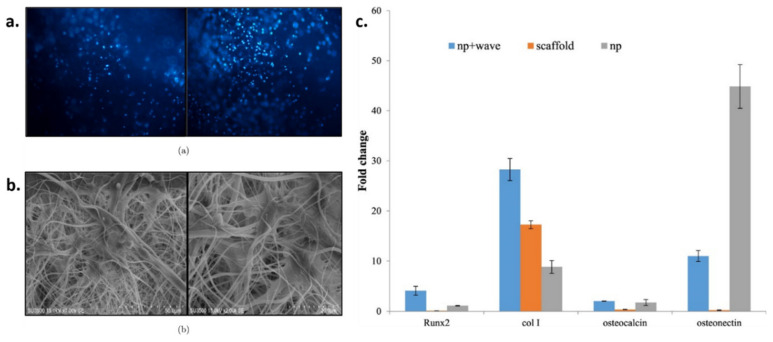
DAPI stained hMSCs 3 days post seeding on CA (left) and CA/rGO-Co (right) scaffolds (**a**) SEM images of the stem cells seeded on CA (left) and CA/rGO-Co (right) nanofibrous scaffolds (**b**) Relative expressions of Run × 2, Col1, OCN and OC genes for hMSCs cultured on CA and CA/rGO-Co, with (np + wave) or without (np) AMF (**c**) [101]. Reproduced with copyright permission.

Liu et al. incorporated GO in CA solutions in acetone/dimethyl formamide (DMF) and electrospun the resulting mixture to obtain hybrid CA/GO nanofibrous mats. The composites presented a uniform and smooth surface and a decreased fiber diameter compared to neat CA. The Raman spectrum of CA/GO showed characteristic peaks at 1300 cm^−1^ (graphene D band) and 1580 cm^−1^ (graphene G band), with increased intensity at higher GO contents. The incorporation of GO into the CA fibers improved their mechanical properties, a higher strain at break and an increased Young’s modulus being observed in the stress-strain curves of the composites, compared to neat CA. To investigate the cellular adhesion behavior, human mesenchymal stem cells (hMSCs) were cultured for 1, 2, 4 and 8 h on CA/GO and the results were compared with the control group represented by cells grown on conventional tissue culture polystyrene (TCP) substrates. As expected, hMSCs adhered more efficiently onto the hybrid scaffolds, most likely due to their increased hydrophilicity and high surface area that creates a good environment for cellular retention. The nanofibrous scaffolds were immersed in simulated body fluid (SBF) and the biomineralization process was observed by SEM. The production of calcium phosphate increased proportionally to the SBF incubation time and the concentration of GO in the nanofibers. It is well known that biomineralization in hybrid materials is governed by the availability of functional groups [100]. In this case, the hydroxyl and carboxyl functional groups in facilitated the deposition of Ca^2+^ ions and facilitated the formation of apatite crystals on the nanofibrous mats. HMSCs seeded on GO-CA substrates also showed significantly higher alkaline phosphatase activity than the control group, in differentiation medium [94]. In another study, reduced graphene oxide-cobalt composite nanoparticles were incorporated in CA-based electrospun scaffolds to investigate their potential to enhance human mesenchymal stem cells (hMSCs) osteogenic differentiation under alternative magnetic field (AMF). SEM images showed that the copper nanoparticles were well attached and evenly decorated the surface of the rGO sheets. The cellular biocompatibility of the electrospun mats was confirmed by MTT assay and 4′,6-diamidino-2-phenylindole (DAPI) nucleus staining performed on the seeded hMSCs (Figure 6a). Even if cellular adhesion was similar for both hybrid and neat CA fibers, the cell growth orientation on CA/rGO-Co was improved (Figure 6b). Moreover, certain genes associated with novel bone formation (e.g., Runx2, OC, Col 1, OCN) presented an enhanced activity when the hybrid scaffolds were exposed to AMF (Figure 6c) [101]. 

This study opens up new possibilities for the development of magnetic cellulose-based composites with graphene, besides its demonstrated bioactivity, the carbonaceous structure also being an appropriate support for the immobilization of magnetic nanoparticles.

The development of scaffolds for tumor cell culture is another area of application for nanofibrous CA/GO hybrids. These in vitro models are essential for the evaluation of cytostatics prior to their introduction on the market and also for the study of cancer cells biological features. In a recent study conducted by Wan et al., electrospun CA/GO scaffolds, seeded with human breast cancer cells (MCF-7), were investigated. Cellulose acetate solutions were prepared by dissolving CA in a mixed solvent system of acetone/acetic acid/dichloromethane with a volume ratio of 2/2/1. Subsequently, a GO suspension was added into the CA solution and the system was kept under magnetic stirring for 5 h at room temperature. After vacuum drying, the morphology of the obtained materials was observed using SEM microscopy. It was found that the addition of GO does not significantly influence the scaffold morphology except for the fact that the fibers diameters decreased. The presence of GO and its interactions with the CA matrix were confirmed by the presence of specific D and G bands in the structure of CA/GO, corresponding to ordered sp2 bonded graphitic carbon of GO, and also by the obvious OH peak shift toward lower values in the FTIR spectra of the composites, due to the formation of hydrogen bonds between CA and GO. MCF-7 cell adhesion, viability and proliferation were assessed using live staining with fluoresceindiacetate, rhodamine phalloidin and 4′,6-diamidino-2-phenylindole, followed by observation under fluorescence microscopy. The results revealed a progressive increase in the number of viable cells for both CA and CA/GO scaffolds; however, at the end of the 5 days testing period, there were fewer cells on the neat CA scaffolds compared to CA/GO. These results confirm that GO had a positive influence on the ability of CA scaffolds to sustain MCF-7 cell development [102]. In another study performed by the same research group, electrospun CA/GO microfibrous hybrids were placed on previously synthesized BC pellicles and culture medium was sprayed over them to initiate the in situ BC synthesis inside the CA/GO mats. The layered material was purified by boiling in NaOH solution and washed several times with distilled water until a neutral pH was reached. SEM images revealed the interpenetrated structure of the BC/CA/GO scaffolds. Two different kind of fibers with average diameters of 43.5 nm and 2.2 µm were identified. The purpose of combining nanofibers with microfibers was to obtain a more intricate ECM-like environment, which may notably improve cell-cell and cell-ECM communication. GO was hardly visible in the SEM images because the carbonaceous sheets were embedded in the polymeric matrix, still, the OH peak shift in the FTIR spectrum and the existence of D and G bands at 1345 and 1590 cm^−1^ in the Raman spectra confirmed its presence in the structure of the composites and its interactions with the cellulosic matrix. Biological characterization by CCK-8 and live cell staining procedures indicated that the cancer cell spreading and proliferation was dependent on the GO content, better results being obtained for the GO-incorporated scaffolds compared to neat CA/BC [103].

Phase inversion is a well-established method for the production of cellulose acetate membranes with applications in biomedical engineering and water purification. The process consists of casting the polymer solution on a proper substrate and immersion in a coagulation bath containing a non-solvent to precipitate the membrane. Membranes obtained by this procedure have different structural characteristics than electrospun mats. They are usually asymmetric, consisting of dense layer in top and a porous substructure at the bottom [90]. Ignat et al. used phase inversion to developed cellulose acetate membranes reinforced with graphene oxide and carbon nanotubes (CNT). The polymer was dissolved in N,N′-dimethylformamide under constant stirring and a small amount of NaOH was added to increase the concentration of surface hydroxyl groups for a better polymer-filler compatibility. The carbonaceous fillers were effectively dispersed in the organic matrix by ultrasonication. The membranes were obtained by casting the CA/GO-CNT solution on a glass slide and immersing it in a coagulation bath containing 2-propanol and distilled water. Micro CT analysis showed that CA/GO-CNT membranes exhibited a porous morphology, with open and interconnected porosity, appropriate for cellular migration within the material. The ability of CA/GO-CNT membranes to guide human adipose derived stem cells (hASCs) differentiation toward the adipogenic lineage was evaluated using Oil Red O staining. A higher accumulation of intracellular lipids was observed on the CA/GO-CNT-grown cells, compared to neat CA after 7 and 21 days. Also, a more pronounced Perilipin gene expression was observed in the CA/GO-CNT hybrids compared to the CA reference. Alizarin Red S staining was employed to evaluate the capacity of CA/CNT-GO membranes to induce osteogenesis in hASCs. Neat CA-cultured cells became round, this indicating that osteogenesis was initiated but no further changes were detected during the 21 days test period. In the case of the CA/CNT-GO membranes, the mineralization levels increased proportionally to the content of GO and CNTs. The expression of osteopontin (OPN) was evaluated via qPCR. It was that found that after 21 days, the gene activity was gradually increased on both neat CA and hybrid membranes but in a higher measure for CA-CNT/GO [54]. According to the results of this study, GO and CNT have the ability to selectively improve hASCs differentiation and could be used for the design of novel materials for well-defined tissue engineering applications.

**Figure 7:**
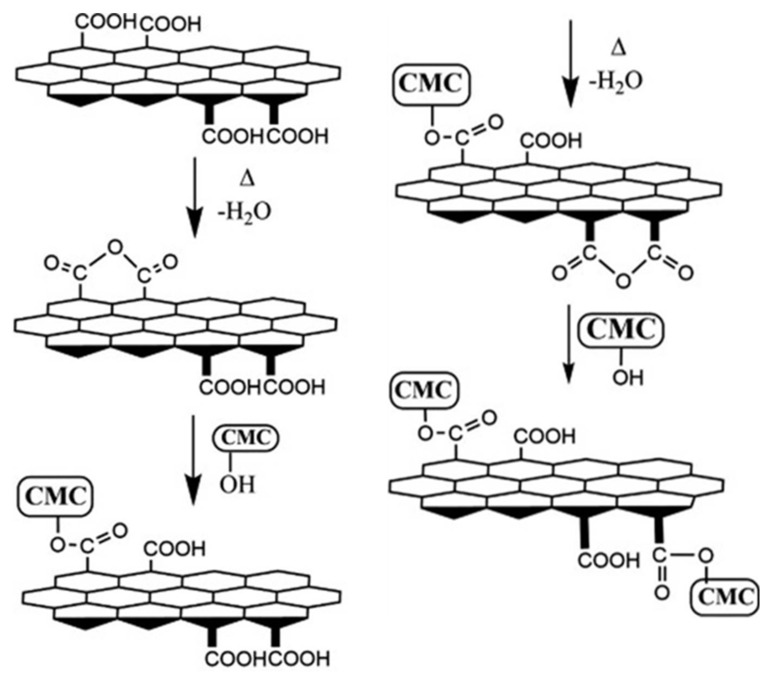
Schematic illustration of the proposed formation mechanism for CMC/GQDs hydrogels [104]. Reproduced with copyright permission.

Hydrogels are another category of promising materials for tissue engineering, their soft and highly hydrated structure resembling the ECM of native tissues. Crosslinkers are essential components for the preparation of hydrogels, since the crosslinking process improves the mechanical properties and degradation rate of these materials. The disadvantage of most crosslinkers is their cytotoxicity and poor biodegradability, characteristics that make them inappropriate for biomedical applications [38]. Citric acid-derived graphene quantum dots (GQDs) were employed as safe, biocompatible, crosslinking agents for carboxymethyl cellulose (CMC). GQDs, CMC and glycerol as plasticizing agent were dissolved in distilled water using magnetic stirring. The resulting paste was cast to a polystyrene plate and cured at 60 °C for 24 h to obtain a hydrogel film with thickness of approximately 10 µm. The proposed hydrogel formation mechanism is represented in Figure 7. The carboxylic groups present on GQDs surface dehydrates, thus forming a cyclic anhydride that further reacts with the hydroxyl groups of CMC chains, forming an ester linkage. The reaction continues until the hydrogel is crosslinked via esterification [104].

The drug delivery ability of the CMC/GQDs hydrogels was studied using doxorubicin (DOX) as a model anticancer drug. It was found that the composites released DOX in a pH-dependent manner, most likely due to the pH sensitive swelling of CMC. The hydrogels did not swell in simulated acidic medium (pH 2), thus protecting the loaded drug from degradation at the stomach level, while in the pH range of 4–8.5 corresponding to the duodenum, the highest swelling and drug release were recorded. The swelling and degradation processes also lead to the release of small material fragments. Their potential cytotoxicity on human colon adenocarcinoma HT29 cells was studied by MTT assay and it was found that cellular viability was over 80% even at high concentrations of GQDs (45%) [104,105]. 

Graphene oxide is a valuable candidate for the development of smart drug delivery systems, its large surface area and oxygenated edge groups ensuring it a high drug loading capacity and potential for further functionalization. Moreover, it was found that due to its amphiphilic character, GO is able to stabilize hydrophobic drugs [106]. Several researchers developed GO-based controlled drug delivery systems for cancer treatment [107,108,109,110]. Jiao et al. used carboxymethylcellulose-grafted graphene oxide (CMC/GO) loaded with methotrexate (MTX) to obtain a pH sensitive drug delivery system for colon cancer therapy. CMC was grafted on GO via ethylene diamine by hydrothermal treatment at 90 °C for 10 h. MTX was dissolved in NaOH and predetermined quantities of freeze dried CMC-GO were added under ultrasonication to obtain various drug loading percentages. The drug release rate ranged from 4.76% in simulated buffer solution (SBF) with pH 1 to 67.4% in SBF with pH 7.4. The differences were related to the higher swelling of CMC at basic pH due to the protonation of –COO^-^ groups. The CMC/GO composites presented a negligible cytotoxicity against NIH3T3 cells during MTT assays and the cellular viability was better for the CMC/GO-MTX-treated cells compared to free MTX-treated ones. Metastatic tumor models were created by splenic injection of HT-29 cells to female Balb/c mice. The mice were treated for 5 days with MTX and CMC/GO-MTX administered intra-gastric. Tumor growth was evaluated by Hematoxylin-Eosin staining. A superior tumor inhibition activity was observed for CMC/GO-MTX (83.3%) compared to free MTX (72.2%). Additionally, CMC/GO-MTX-treated mice presented reduced liver metastasis and prolonged survival time [108]. Cancer cells often present an overexpression of the folate receptor, a membrane glycoprotein considered a highly selective tumor marker. The folate receptor binds folic acid with high affinity [111]. Based on this phenomenon, Sahne et al. designed folate-targeting drug delivery systems based on carboxymethylcellulose, polyvinylpyrrolidone (PVP) and spherical graphene oxide nanoparticles synthesized through carbon rehybridization, chemical exfoliation and centrifugation. CMC was modified by thiolation whereas PVP was enriched with amine and thiol-reactive end groups by reversible addition-fragmentation chain transfer (RAFT) polymerization. The functionalized polymers were deposited layer by layer on the surface of GO. Curcumin (Ccm), a polyphenol with antioxidant and antitumoral properties was encapsulated in the CMC layer during the deposition process and monoclonal folic acid antibodies (FA) were grafted on the CCm/CMC-PVP-GO using polyethylene glycol (PEG) as linker. Cytotoxicity studies were performed on MCF7 and Saos 2 cells and 4T1 bearing Balc/c mice were used as tumor models. The Ccm-FA/CMC-PVP-GO presented an inhibition rate of 76 and 81% against Saos 2 and MCF7 tumor cell lines and a 76% antitumor efficiency, expressed by antiangiogenesis, apoptosis and tumor growth inhibition in vivo [109]. 

### 2.4. Cellulose Nanocrystals-Graphene Composites

**Figure 8:**
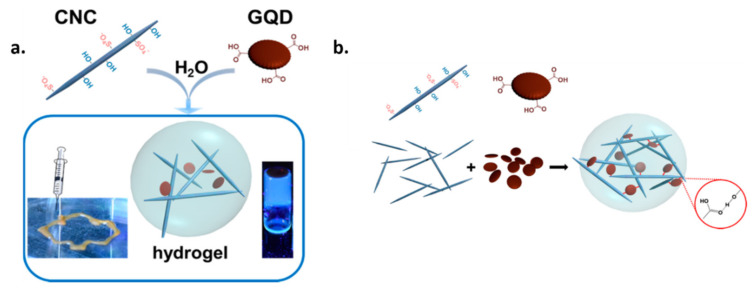
Schematic representation of the CNCs and GQDs hydrogels building blocks and illustration of the shear thinning behavior and photoluminescence properties (**a**) Hydrogen bonds formation between GQDs and CNCs upon mixing (**b**) [112]. Reproduced with copyright permission.

A special type of cellulose-based hydrogels were prepared by Khabibullin et al. using cellulose nanocrystals (CNCs) as building blocks [112]. The characteristics that determined the choice of CNCs for this application are their widespread availability, high mechanical properties, native biocompatibility, and facile surface functionalization. Graphene quantum dots were used as crosslinkers for the CNC-based hydrogels, their addition not only reinforcing the cellulosic structure but also providing it fluorescence properties. The GQDs were dispersed in aqueous solutions of CNCs and the suspensions were vortex mixed for 15 s. The formation of CNC/GQD network was governed by the interactions between the surface hydroxyl and half ester groups on CNCs surface and the carboxyl moieties on GQDs edges (Figure 8b). Hydrophobic interactions could also occur between CNCs hydrophobic faces and GQDs basal planes.

The CNC/GQD hydrogels exhibited a shear thinning behavior during rheological evaluation. At 1% strain, the hybrid suspension formed a hydrogel with G = 80 Pa while at 50% strain, the value of G’ decreased to 2 Pa, this signifying gel liquefaction. To exploit this characteristic, the hydrogels performance as injectable material was examined using a 3D printer. The printed hydrogel threads retained their structure and were able to create predetermined patterns. Also, they exhibited variable photoluminescence in the spectral range of 400–680 nm when excited at 365 nm (Figure 8a) [112]. The physico-chemical characteristics of these materials suggest that they could be used as injectable composites for tissue engineering applications, particularly for bioimagistics and biosensing. However, a thorough biocompatibility assessment should also be performed to demonstrate that their use in the biomedical field is risk-free.

In another study conducted by Kumar et al., GO nanosheets and CNCs were employed as multifunctional crosslinking agents for polyacrylamide-sodium carboxymethyl cellulose (PAA-NaCMC) hydrogels, prepared by in situ free radical polymerization. Due to GO and CNCs hydrophilic nature and ability to provide sites for hydrogen and covalent bonding, both PAA and NaCMC had favorable molecular interactions with GO and formed strong interfacial bonds with CNCs. The synergistic effect of GO and CNC lead to an improvement of the viscoelastic mechanical properties, shape recovery behavior and self-healing ability of the interpenetrating PAA/NaCMC network. Still, further studied must be conducted in order to determinate if these hybrid hydrogels represent appropriate 3D microenvironments for tissue engineering applications [38].

**Figure 9:**
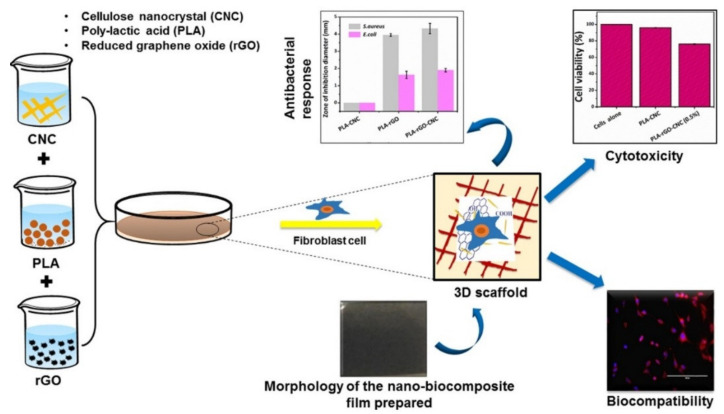
Schematic description of the method used for the preparation of nanocomposite films (**left**); Antibacterial properties, cytotoxicity, biocompatibility, and morphology of the PLA/CNC-rGO composites (**right**) [61]. Reproduced with copyright permission.

Polylactic acid is a biobased plastic obtained by bacterial fermentation processes. Its biodegradability and biocompatibility recommend it for replacing conventional petroleum-based plastics used in the biomedical field. Still, its mechanical properties need further improvement. CNCs and rGO were used as reinforcing fillers for PLA [61,62]. The polymer was dissolved in chloroform and a chloroform suspension of CNCs and rGO was gradually added into the polymeric solution under vigorous magnetic stirring. The mixture was casted on circular Petri plates and left to dry at room temperature (Figure 9 (left)). The traces of solvent were removed by further drying the PLA films in a laboratory oven, at 40 °C for 3 h. The filler addition increased the tensile strength but also slightly decreased the ductility, thus resulting a lower elongation at break. The biocompatibility of the PLA films was evaluated by seeding NIH3T3 fibroblasts on their surface and observing the changes in cellular morphology 24 h post seeding via fluorescence microscopy (Figure 9 (right)). Minor changes such as small cytoplasmatic lesions were observed but they were not considered a sign of cytotoxicity. An interesting fact was that rGO induced an antibacterial character to the PLA films, as observed during the disk diffusion assay. The samples containing only 0.5 wt % rGO presented an antibacterial activity that was not noticed on neat PLA or PLA/CNC composites (Figure 9 (right)) [61]. 

This study suggests that rGO is not only a reinforcing agent for biopolymer matrices but can also induce an antimicrobial character, very useful for preventing infections in tissue engineering applications.

Undeniably, cellulose and graphene are highly versatile materials and they can be employed in multiple ways to obtain hybrid composites with superior physico-chemical and biological features compared to cellulose or graphene alone. A summary of the cellulose/graphene composites for tissue engineering applications presented in the previous chapters is found in Table 1. 

## 3. Conclusions and Future Perspectives

This review highlighted some of the recent discoveries regarding cellulose-based composites with graphene and graphene derivatives. As most of the described studies concluded, the combination of cellulose and graphene renders sustainable and cost effective composites with improved physico-chemical features and bioactivity. Cellulose and graphene can be labeled as complementary materials because the incorporation of graphene in cellulose-based matrices minimizes GE’s potential cytotoxic character and, in its turn, GE improves the biocompatibility of cellulose by providing cell binding sites and positively interfering in cellular differentiation processes and also its mechanical properties. The versatility of both cellulose and graphene is another important reason cellulose/graphene composites are needed in tissue engineering. Cellulose paper was used to develop electrodes for cellular electrical stimulation or in vivo tissue models for a better understanding of a specific tissue biology and experimental drug testing. Bacterial cellulose and cellulose derivatives were employed in the form of electrospun mats, membranes, and hydrogels as ECM-mimicking scaffolds for enhanced cell adhesion, growth and proliferation whereas cellulose nanocrystals and cellulose nanofibers were incorporated in various biopolymer matrices for a reinforcing effect. Some particularly interesting features for tissue engineering are related to GE and its derivatives ability to guide stem cells differentiation toward specific lineages (osteogenic, adipogenic, angiogenic) and also to the electrical conductivity and fluorescent properties that they provide to cellulose matrices, thus extending the areas of application of cellulose/graphene composites Also, a synergistic reinforcing effect was observed when both cellulose and graphene-based fillers were incorporated in various biopolymer matrices.

The constant research activity in the field of cellulose/graphene composites lead so far to important discoveries in the area of tissue engineering. Even if these materials could represent promising alternatives to currently used medical techniques, the progress toward obtaining clinical products is slow. Future trends consist of the collaboration of clinicians, biomaterial scientists and engineers, with expertise in their own fields, to elaborate marketable and accessible products for therapeutic purposes.

## Figures and Tables

**Table 1:** Components, preparation methods and applications of the cellulose/graphene composites presented in this review.

Cellulose Type	Graphene Type	Composite Preparation Method	Application	Ref.
Cellulose paper (commercial, tissues, blotting paper, filter paper)	rGO	Drop-wise deposition of GO (aqueous dispersion) on the paper substrate and GO reduction with L-ascorbic acid followed by lamination of the G-C papers with alginate Integration of the G-C papers in polystyrene chambers	Multilayered constructs for bone tissue engineering Electrodes for concomitant cell culture and electrical stimulation	[58,59]
BC	GE, GO	Membrane-liquid interface culture (MILIC)	Cell culture scaffolds	[87]
BC	rGO	In situ biosynthesis and bacteria-mediated reduction	Cell culture scaffolds with electrical stimulation potential, biosensors	[89]
BC	GO-HA	Impregnation of the wet BC pellicle with an ethanolic GO-HA suspension	Cell culture scaffolds for bone tissue engineering	[91]
BC	GO	In situ biosynthesis	Cell culture scaffolds for neural tissue engineering	[86]
BC	GO stabilized with APCLP	Non-genetic manipulation of Acetobacter xylinum	E18 neurons culture scaffolds used to construct a 3D neuronal network (minibrain)	[93]
BC	GO covered with APCLP	In situ biosynthesis	Brain cortex mimetics	[92]
CA	GO	Electrospinning	Cell culture scaffolds for bone tissue engineering	[94]
CA	rGO-Co	Electrospinning	Cell culture scaffolds for enhances osteogenic differentiation under alternative magnetic field (AMF)	[101]
CA	GO	Electrospinning	Tumor cell culture scaffolds for	[102]
BC/CA	GO	Electrospinning of CA/GO solution and impregnation with BC culture medium for in situ biosynthesis	Tumor cell culture scaffolds with improved ECM-like features	[103]
CA	GO-CNT	Phase inversion	Membranes for guided hASCs differentiation	[54]
CMC	GQDs	Crosslinking of aqueous CMC suspension by GQDs via esterification	ECM-like scaffolds with pH sensitive drug delivery potential	[104,105]
CMC	GO	Grafting of CMC on GO via hydrothermal treatment	pH sensitive drug delivery systems for colon cancer treatment	[108]
CMC	GO	Layer by layer deposition of CMC and PVP on GO nanoparticles, encapsulation of curcumin in the CMC layer, surface grafting of folic acid antibody using PEG as linker	Folate-targeting drug delivery systems for cancer treatment	[109]
CNCs	GQDs	Crosslinking of CNCs aqueous suspensions by GQDs	Injectable hydrogels with photoluminescence properties	[112]
CNCs	GO	Mixing of CNCs and GO in distilled water	Multifunctional crosslinking agents for PAA/NaCMC hydrogels	[38]
CNCs	rGO	Mixing of CNCs and rGO in chloroform	Reinforcing fillers with antibacterial properties for PLA	[61,62]
